# Supplementary material for: Motivating Personal Climate Action through a Safety and Health Risk Management Framework
Source: Int J Environ Res Public Health. 2022 Dec 20;20(1):7. doi: 10.3390/ijerph20010007 (PMC9819491; doi:10.3390/ijerph20010007)
Supplement: Supplementary file 1 [file ijerph-20-00007-s001.zip › ijerph-2095978-supplementary.pdf]

Survey Items

QA-H Please indicate your agreement/disagreement with the questions below	Strongly disagree (1)	Disagree (2)	Neither agree nor disagree (3)	Agree (4)	Strongly Agree (5)
A. Free markets- non-government programs- are the best way to supply people with things they need. (1)	○	○	○	○	○
B. The government interferes far too much in our everyday lives. (2)	○	○	○	○	○
C. People should be able to rely on the government for help when they need it. (3)	○	○	○	○	○
D.It's society's responsibility to make sure everyone's basic needs are met. (4)	○	○	○	○	○
E. It seems like the criminals and welfare cheats get all the benefits, while the average citizen picks up the tab. (5)	○	○	○	○	○
F. We have gone too far in pushing equal rights. (6)	○	○	○	○	○
G. We need to dramatically reduce inequalities between the rich and the poor, whites and people of color, and men and women. (7)	○	○	○	○	○
H. Our society would be better off if the distribution of wealth was more equal (8)	○	○	○	○	○

Q1 Please indicate your level of agreement with the following statement where 100 is strongly agree and 0 is not at all:

	0	10	20	30	40	50	60	70	80	90	100
Climate change is primarily human caused											

Q2 Please indicate your level of agreement with the following statement where 100 is strongly agree and 0 is not at all:

	0	10	20	30	40	50	60	70	80	90	100
Climate change affects me ()											

Q3 Please indicate your level of agreement with the following statement where 100 is strongly agree and 0 is not at all.

	0	10	20	30	40	50	60	70	80	90	100
Humans can resolve the climate crises ()											

Q4 Rank the following climate change issues in order from most to least important. Rank by dragging and dropping the one you believe is most important first and arrange in order from most important (on top) to least important (on bottom).

Climate change is a/n ___________ issue.

_______ scientific issue
_______ environmental issue
_______ ethical/moral issue
_______ political issue
_______ health and safety issue
_______ agricultural issue
_______ economic issue

Q5 Please indicate your level of agreement with the following statement where 100 is strongly agree and 0 is not at all.

	0	10	20	30	40	50	60	70	80	90	100
I am ready to act now to mitigate (prevent) climate change ()											

Q6 What are you doing to mitigate (prevent) climate change now? Select all that apply:

- ▢ Buy less stuff that I don’t need
- ▢ Use less stuff and reduce and reuse items wherever I can
- ▢ Buy locally produced or low carbon alternative products and food
- ▢ Grow/produce some of my own food
- ▢ Change my light bulbs to LED
- ▢ Conserve energy
- ▢ Use more low carbon forms of transportation
- ▢ Buy/drive a zero emissions vehicle (electric car)
- ▢ Install solar panels on my home
- ▢ Install geothermal energy system on my home
- ▢ Go 100% renewable for all my energy consumption needs
- ▢ Speak with my employer to get them to take actions to reduce their carbon footprint
- ▢ Speak with family, colleagues and friends about reducing their carbon footprint
- ▢ Speak with my local elected representatives, (municipal, state/provincial, and federal) about my desire for climate change action
- ▢ none of these

Q7 What do you plan on doing in the <u>future</u> to mitigate (prevent) climate change? Select all that apply:

- ☐ Buy less stuff that I don’t need
- ☐ Use less stuff and reduce and reuse items wherever I can
- ☐ Buy locally produced or low carbon alternative products and food
- ☐ Grow/produce some of my own food
- ☐ Change my light bulbs to LED
- ☐ Conserve energy
- ☐ Use more low carbon forms of transportation
- ☐ Buy/drive a zero emissions vehicle (electric car)
- ☐ Install solar panels on my home
- ☐ Install geothermal energy system on my home
- ☐ Go 100% renewable for all my energy consumption needs
- ☐ Speak with my employer to get them to take actions to reduce their carbon footprint
- ☐ Speak with family, colleagues and friends about reducing their carbon footprint
- ☐ Speak with my local elected representatives, (municipal, state/provincial, and federal) about my desire for climate change action
- ☐ none of these

Q8 What stops you from taking action to prevent climate change? Rank by dragging and dropping the one you believe is most important first and arrange in order from most important on top (#1) to least important on bottom (#5).

______ I'm not sure what I can do that will make a difference
______ I can't afford the carbon free or carbon neutral alternative right now
______ Alternatives do not exist or are not practical for my lifestyle
______ I don't think my family/social group/community group would approve
______ I have a conflict of interest in taking climate change action due to my employment, social or political affiliation.

Q9 Rank these motivators for you to act to mitigate climate change with the most important motivator ranked as #1 (on top) and least is # 3 (on bottom).

______ To protect the health and safety of current and future generations
______ To protect ecosystems and wildlife
______ To protect the economy

Q10 Are you aware of the impacts of climate change? Please check all boxes you think apply: **Climate change will:**

- ▢ result in an increase in severe droughts (1)
- ▢ result in an increase in severe floods (2)
- ▢ result in an increase in wildfires (3)
- ▢ result in an increase in severe weather events (4)
- ▢ cause food shortages and famine (5)
- ▢ cause more vector borne illness and fatalities (6)
- ▢ cause more heat related illness and fatalities (7)
- ▢ cause large parts of the Earth to become uninhabitable (8)
- ▢ cause millions of people to become displaced from their homes (9)
- ▢ result in an increase in violent conflict (10)
- ▢ cost trillions of dollars in property damage/loss (11)
- ▢ have a negative effect on the economy (12)
- ▢ have a negative effect on human health and safety (13)

Page Break

Video Please watch this entire video. Do not fast forward.

Page Break

Q11 Now that you've watched the video, please indicate your level of agreement with the following statement where 100 is strongly agree and 0 is not at all:

	0 10 20 30 40 50 60 70 80 90 100
Climate change is primarily human caused ()	

Q12 Please indicate your level of agreement with the following statement where 100 is strongly agree and 0 is not at all:

	0 10 20 30 40 50 60 70 80 90 100
Climate change affects me ()	

Q13 Please indicate your level of agreement with the following statement where 100 is strongly agree and 0 is not at all:

	0 10 20 30 40 50 60 70 80 90 100
Humans can resolve the climate crises ()	

Q14 Rank the following climate change issues in order from most to least important. Rank by dragging and dropping the one you believe is most important first and arrange in order from most

important (on top) to least important (on bottom).

Climate change is a/n ___________ issue.

______ scientific issue
______ environmental issue
______ ethical/moral issue
______ political issue
______ health and safety issue
______ agricultural issue
______ economic issue

Q15 Please indicate your level of agreement with the following statement where 100 is strongly agree and 0 is not at all.

	0 10 20 30 40 50 60 70 80 90 100
I am ready to act now to mitigate (prevent) climate change ()	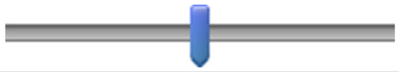

Q16 What stops you from taking action to prevent climate change? Rank by dragging and dropping the one you believe is most important first and arrange in order from most important on top (#1) to least important on bottom (#5).

______ I'm not sure what I can do that will make a difference
______ I can't afford the carbon free or carbon neutral alternative right now
______ Alternatives do not exist or are not practical for my lifestyle
______ I don't think my family/social group/community group would approve
______ I have a conflict of interest in taking climate change action due to my employment, social or political affiliation.

Q17
Rank these motivators for you to act to mitigate climate change with the most important motivator ranked as #1 (on top) and least is # 3 (on bottom).

______ To protect the health and safety of current and future generations
______ To protect ecosystems and wildlife
______ To protect the economy

Q18 Are you aware of the impacts of climate change? Please check all boxes you think apply: **Climate change will:**

- ▢ Result in an increase in severe droughts (1)
- ▢ Result in an increase in severe floods (2)
- ▢ Result in an increase in wildfires (3)
- ▢ Result in an increase in severe weather events (4)
- ▢ Cause food shortages and famine (5)
- ▢ Cause more vector borne illness and fatalities (6)
- ▢ Cause more heat related illness and fatalities (7)
- ▢ Cause large parts of the Earth to become uninhabitable (8)
- ▢ Cause millions of people to become displaced from their homes (9)
- ▢ Result in an increase in violent conflict (10)
- ▢ Cost trillions of dollars in property damage/loss (11)
- ▢ Have a negative effect on the economy (12)
- ▢ Have a negative effect on human health and safety (13)

Q19 What actions are you willing to take in the next 0-90 days? Select all that apply:

- ☐ Buy less stuff that I don't need
- ☐ Use less stuff and reduce and reuse items wherever I can
- ☐ Buy locally produced or low carbon alternative products and food
- ☐ Grow/produce some of my own food
- ☐ Change my light bulbs to LED
- ☐ Conserve energy
- ☐ Use more low carbon forms of transportation
- ☐ Buy/drive a zero emissions vehicle (electric car)
- ☐ Install solar panels on my home
- ☐ Install geothermal energy system on my home
- ☐ Go 100% renewable for all my energy consumption needs
- ☐ Speak with my employer to get them to take actions to reduce their carbon footprint
- ☐ Speak with family, colleagues and friends about reducing their carbon footprint
- ☐ Speak with my local elected representatives, (municipal, state/provincial, and federal) about my desire for climate change action
- ☐ none of these

Q20 What actions are you willing to take at some time in the future? Select all that apply:

- [ ] Buy less stuff that I don’t need
- [ ] Use less stuff and reduce and reuse items wherever I can
- [ ] Buy locally produced or low carbon alternative products and food
- [ ] Grow/produce some of my own food
- [ ] Change my light bulbs to LED
- [ ] Conserve energy
- [ ] Use more low carbon forms of transportation
- [ ] Buy/drive a zero emissions vehicle (electric car)
- [ ] Install solar panels on my home
- [ ] Install geothermal energy system on my home
- [ ] Go 100% renewable for all my energy consumption needs
- [ ] Speak with my employer to get them to take actions to reduce their carbon footprint
- [ ] Speak with family, colleagues and friends about reducing their carbon footprint
- [ ] Speak with my local elected representatives, (municipal, state/provincial, and federal) about my desire for climate change action
- [ ] none of these

Q21 The information presented has:

- ○ very much improved my perceptions, knowledge, and awareness of climate change and mitigation
- ○ improved my perceptions, knowledge, and awareness of climate change and mitigation
- ○ not changed my perceptions, knowledge and awareness of climate change and mitigation because I already knew this information
- ○ not changed my perceptions, knowledge and awareness of climate change and mitigation for other reasons
- ○ decreased my perceptions, knowledge and awareness of climate change and mitigation
- ○ very much decreased my perceptions, knowledge, and awareness of climate change and mitigation

---

Q22 **The information presented has:**

- ○ very positively changed my motivation to act to mitigate climate change
- ○ positively changed my motivation to act to mitigate climate change
- ○ not changed my motivation to act because I was already motivated to act
- ○ not changed my motivation to act for other reasons
- ○ negatively affected my motivation to mitigate climate change
- ○ very negatively affected my motivation to mitigate climate change

---

Q23 Would you like to learn more about climate change action through the following: Select all that apply

- ☐ workplace
- ☐ community
- ☐ both
- ☐ neither, I do not wish to learn more about climate action
